# Common pulmonary vein atresia: a case report and treatment experience sharing of a newborn diagnosed with common pulmonary vein atresia after two operations

**DOI:** 10.3389/fped.2025.1590620

**Published:** 2025-08-21

**Authors:** Kun Zhang, Shuguang Tao, Wei Gao, Jiangrong Gu, Linlin Wen, Yun Fan

**Affiliations:** Department of Cardiac Surgery, Hebei Children’s Hospital, Shijiazhaung, Hebei, China

**Keywords:** common pulmonary vein atresia, pulmonary lymphangiectasia, pulmonary venous obstruction, pulmonary venous drainage, cardiac surgery

## Abstract

Common pulmonary vein atresia (CPVA) is a rare congenital heart disease characterized by the absence of functional connectivity between the pulmonary vein and any other heart cavity or systemic venous structure. A 13-h-old newborn (G3P3) was admitted to the department of pediatrics of a local maternity hospital and given tracheal intubation ventilator for assisted breathing due to systemic cyanosis, respiratory distress, and poor response 4 h after birth. He was transferred to Handan Maternal and Child Health Hospital 7 h after birth. After entering the department of neonatology, the child was considered to have anomalous pulmonary venous drainage (subcardiac type) after undergoing color Doppler examination. There was no obvious effect on the treatment, such as liquid replenishment and acid correction, so he was transferred to our hospital. After admission, respiratory failure complicated with heart failure, metabolic acidosis, and hyperlactatemia were initially diagnosed. After two-dimensional echocardiography, emergency surgery was decided after consideration of CPVA. Complete pulmonary venous malformation drainage and cardiopulmonary bypass-assisted repair were performed 9 h after admission. The patient was successfully discharged from the ICU on the 30th day after surgery, but on the 52nd day after surgery, pulmonary vein obstruction was found again. After emergency cardiopulmonary bypass-assisted repair, the pulmonary vein obstruction was corrected, but the child had multiorgan failure (MOF), coagulation function was abnormal, and vital signs were still difficult to maintain with cardiopulmonary bypass assistance after the second operation, and the family gave up treatment. Although we have made significant progress in understanding and processing CPVA, the early diagnosis, surgical treatment, and prognosis remain challenging. The changes in examination data we found in the evolution of the patient's condition during this period and the corresponding changes required constant alertness to the possibility of various problems, such as the occurrence of pulmonary vein obstruction and occlusion, and timely targeted treatment strategies. The treatment experience and lessons during this period can be referred to by everyone, hoping to provide some help in improving the treatment of CPVA patients.

## Introduction

Common pulmonary vein atresia (CPVA) is a rare congenital heart disease characterized by the absence of functional connectivity between the pulmonary vein and any other heart cavity or systemic venous structure. There is no outlet or atretic fibrous band from the pulmonary vein to the right atrium, left atrium, or systemic vein (1). CPVA can be classified as complete and partial atresia. The clinical manifestations of CPVA are varied and often lack specificity, which makes early diagnosis challenging. Common symptoms include, but are not limited to, difficulty breathing, stunted growth, frequent respiratory infections, cyanosis (a bluish purple appearance of the skin or mucous membranes), and decreased activity tolerance. As the disease progresses, patients may develop signs of heart failure, such as edema, liver enlargement, and jugular vein irritation.

In 1962, Lucas et al. (2) first reported CPVA and defined it as the lack of connection between the pulmonary vein junction and the heart or any major vein in the body (3). By searching foreign literature data, we found that as of 2008, according to Vaideeswar et al. (4), 25 cases of CPVA were recorded. By 2015, according to Perez et al. (3), this number had increased to 35 cases. However, among these <40 cases, only 5 cases were successfully treated by surgery, with a significant adverse prognosis (5–7). At present, only seven reports have been retrieved in China, of which three cases involved infants diagnosed with pulmonary lymphangiectasis and four cases of fetal autopsy findings. It has been demonstrated that children with CPVA experience severe complications following birth, often manifested as cyanosis, respiratory and circulatory failure, and acidosis, with a poor prognosis, a high mortality rate, and a low survival rate exceeding 4 weeks (8). In recent years, advancements in the understanding of CPVA and improvements in surgical techniques have led to a few cases achieving survival through effective treatment.

## Case

**Figure 1:**
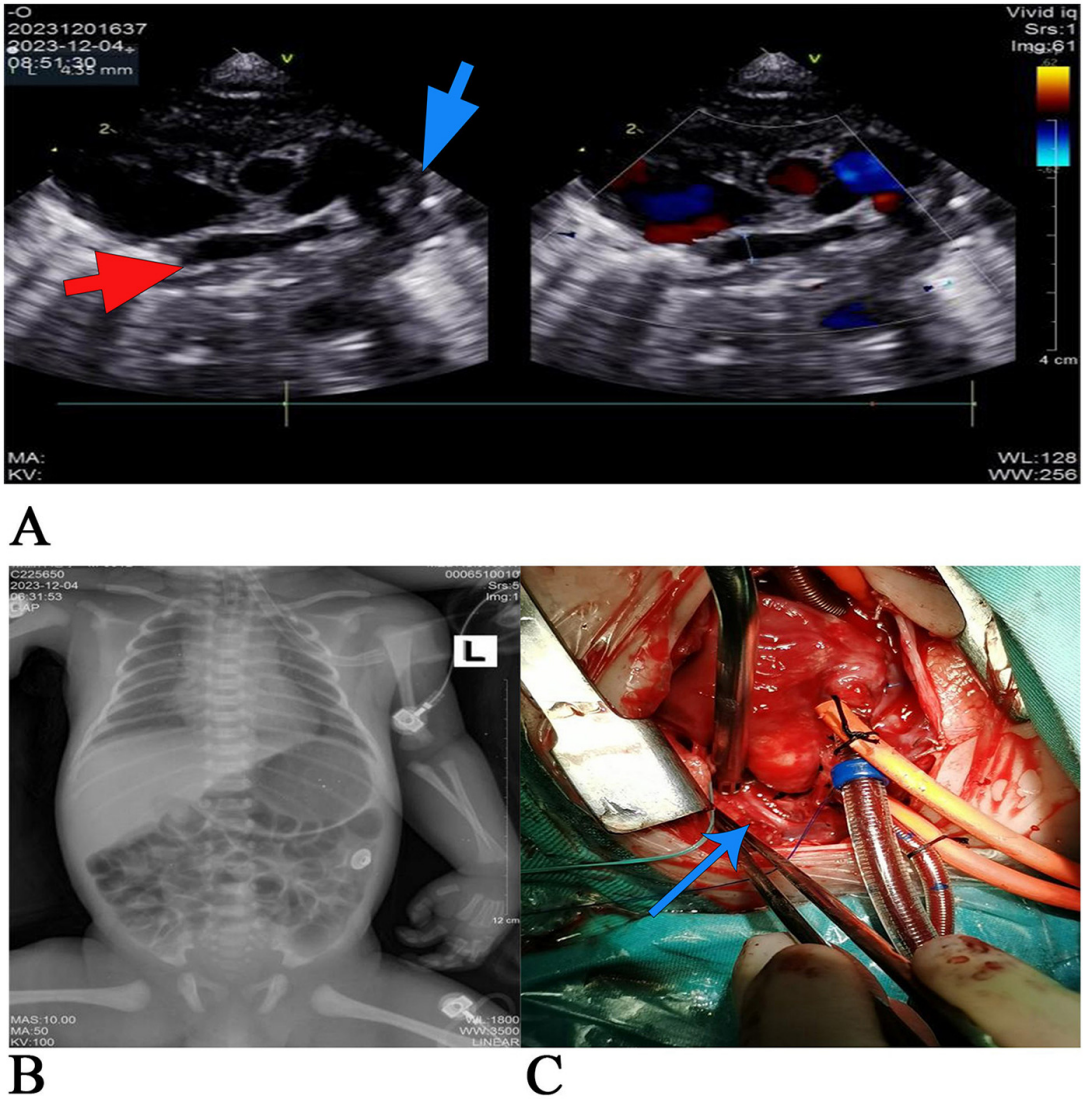
**(A)** The preoperative two-dimensional echocardiography. The red arrow marks the pulmonary venous confluence, and the blue arrow marks vertical veins. The main pulmonary artery was dilated, none of the four pulmonary veins were connected to the left atrium, and the four pulmonary veins were merged into the common pulmonary vein, which had an internal diameter of approximately 4 mm. Slow blood flow in the vertical veins. The drainage route was not explored and clear (considering: CPVA), the tricuspid valve is not closed, and the left aortic arch can be seen with an open arterial catheter with an internal diameter of approximately 4.5 mm. The venous catheter is open. **(B)** Chest x-rays taken 18 h after birth showed a slightly enlarged heart shadow, adequate blood flow in both lungs, the tracheal intubation tip at the level of the first thoracic vertebra, a narrow aortic junction, and a flat pulmonary artery segment. **(C)** Intraoperative image: the blue arrow marks the atretic vertical veins. All four pulmonary veins were not connected to the left atrium and merged into the common pulmonary vein, which joined the left innominate vein through the vertical vein. The vertical vein stenosis and middle and upper muscular atresia were noted.

A 13-h-old male newborn (G3P3), with a birth weight of 3 kg, was delivered at a local county hospital. Cyanosis was found immediately after birth, which was slightly relieved after giving the child oxygen. After 4 h of birth, skin cyanosis of the whole body had aggravated, and the child was admitted to the pediatric department of the birth hospital. Due to poor response and respiratory distress, the child was given tracheal intubation ventilator to assist his breathing and was transferred to Handan Maternal and Child Health Hospital 7 h after birth. After entering the neonatal department, the child was considered to have anomalous pulmonary venous drainage (subcardiac type) after undergoing color ultrasound examination. He was subsequently admitted to the intensive care unit of our department at 01:17 with tracheal intubation, assisted by a ventilator. On admission, his vital signs were as follows: heart rate (HR), 115 beats/min; respiratory rate (RR), 32 breaths/min; blood pressure (BP), 54/25 mmHg; and percutaneous oxygen saturation of 38% in the right lower limb (ventilator oxygen concentration of 60%). The child had a poor response and severe systemic skin cyanosis. After admission, arterial blood gas analysis revealed the following: pH 7.117, PCO_2_ 65.0 mmHg, PO_2_ 13.7 mmHg, K^+^ 4.58 mmol/L, lactate (Lac) 9.3 mmol/L, base excess (BE) 8.4 mmol/L, hematorict (HCT) 47%, NT-proBNP >35,000 pg/ml, and myoglobin 200.64 ng/ml. The two-dimensional echocardiography (Figure 1A) indicated CPVA, and the chest radiography (Figure 1B) indicated a slight enlargement of the cardiac shadow. The primary diagnosis was respiratory failure complicated with heart failure, metabolic acidosis, and hyperlactatemia. Although enhanced CT could have further clarified the diagnosis of CPVA, the patient's condition was critical and required urgent surgery. Therefore, open heart repair with complete pulmonary venous malformation drainage was performed 9 h after admission despite surgical complications and uncertainties.

During moderate hypothermic cardiopulmonary bypass, no residual upper left cavity was identified, and the arterial duct was ligated. The right atrium was incised, revealing a central atrial septal defect of foramen secundum with a diameter of 10 mm, as shown in the intraoperative image (Figure 1C). The left atrial appendage was incised to the left posterior atrial wall, and a sutureless anastomosis was performed between the left atrial appendage and the peripheral tissue of the common pulmonary vein with a 6-0 PDS suture. The atrial septal defect, approximately 5 mm at its center, was sutured continuously with a correspondingly sized autologous pericardial patch and 5-0 Prolene suture. Spontaneous cardiac recovery after open circulation, in sinus rhythm with a heart rate of 120 beats/min. A 10-0 suture was used to ligate the vertical vein to the left innominate vein. After repeated attempts to stop the machine failed, extracorporeal membrane oxygenation (ECMO) was installed, and delayed chest closure was performed. The operation lasted 6 h and 40 min. Postoperatively, the patient’s BP was 60/45 mmHg, percutaneous oxygen saturation was 90%, HR was 170 beats/min, and RR was 35 breaths/min. Endotracheal intubation, urinary tube, and deep vein catheterization were maintained. Due to the critical condition of the child, the child was transferred directly from the operating room to the ICU.

**Figure 2:**
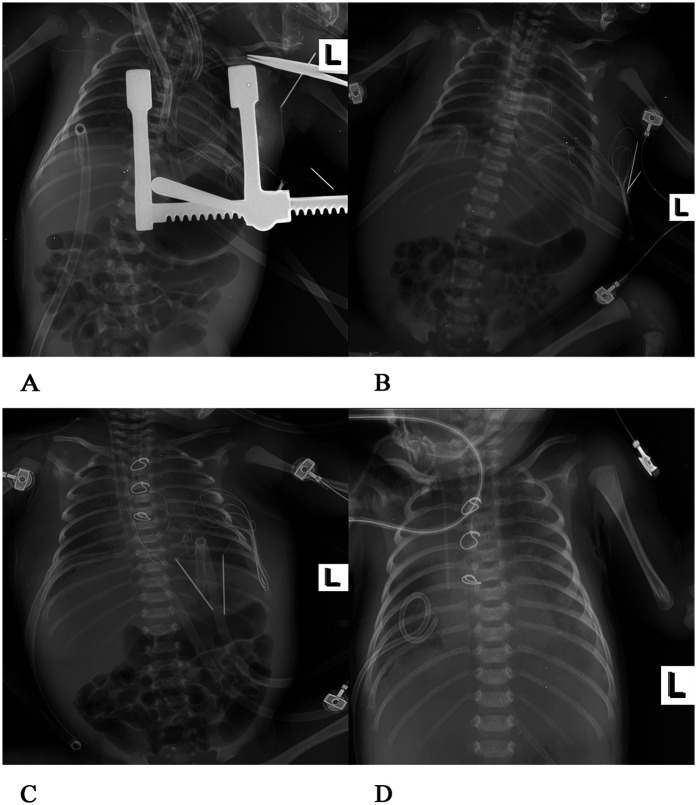
Postoperative chest x-rays at various stages **(A)** 3 h after operation of congenital heart disease, blood in both lungs was normal, there was no consolidation in both lungs, and the shape and size of the heart were normal. **(B)** On the 6th day after surgery, there was a light shadow in both lungs, a small amount of exudation in both lungs, and soft tissue swelling in both thoracic and abdominal walls, and the tip of the tracheal intubation was flat at the upper margin of the second thoracic vertebra. **(C)** On the 8th day after surgery, the transmittance of both lungs was reduced, and flaky-like blurred shadows were visible. A strip of high transmittance was visible in the upper right lung with the edge of compressed lung tissue, a right pneumothorax, a heart of normal shape and size, and the tracheal intubation tip was flat at the third thoracic vertebra. **(D)** On the 23rd day after surgery, a large flake high-density shadow was seen in both lungs, the metal ring was intact, no fracture was observed, both lungs exuded, right upper lung atelectasis, and the heart shape was normal.

**Figure 3:**
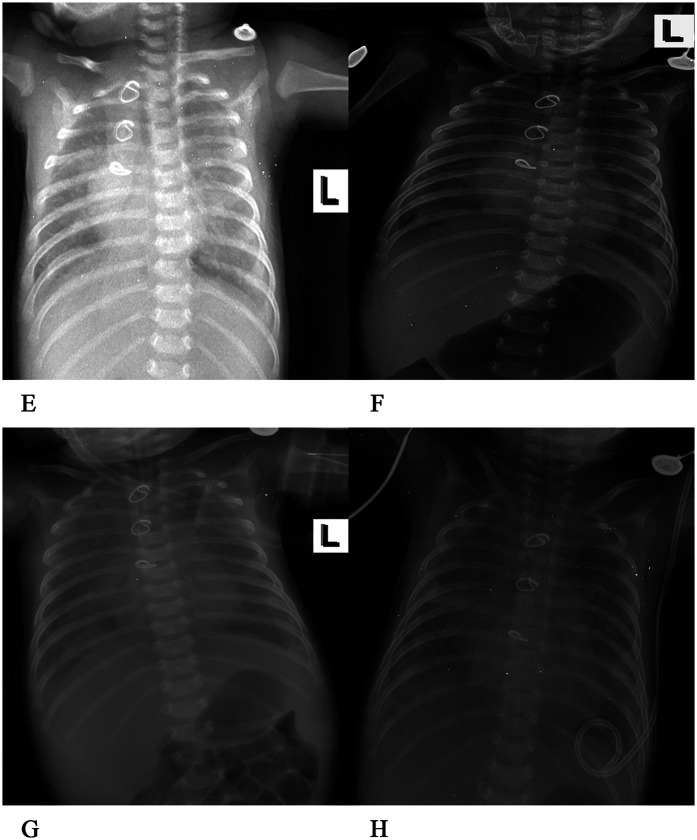
**(E)** On the 35th day after the operation, patchy shadows were visible in both lungs, with exudation. The structure of the metal ring was intact, and the shape and size of the heart were normal. **(F)** On the 39th day after the operation, exudation was present in both lungs, and the bilateral abdominal fat lines were clear. **(G)** On the 46th day after the operation, faint patchy shadows were seen in both lungs, the bilateral cardiac margins were blurred, and the tip of the endotracheal tube was at the level of the fifth thoracic vertebra. **(H)** On the 51st day after the operation, the transparency of both lungs decreased, large areas of blurred shadows were present in both lungs, the shape of the heart was blurred, and the tip of the endotracheal tube was at the level of the fourth thoracic vertebra.

**Figure 4:**
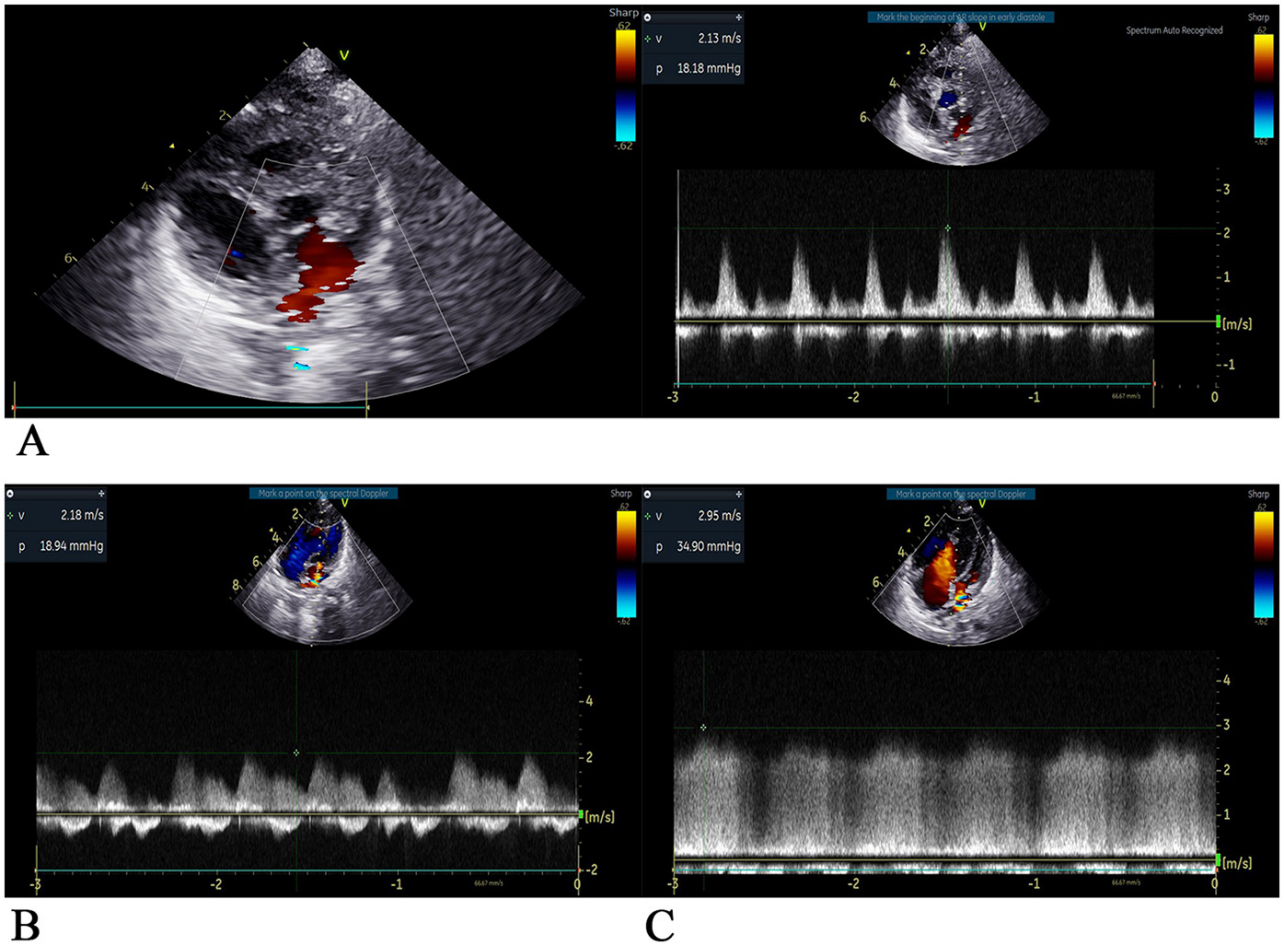
Two-dimensional ultrasound images of the heart at each stage after surgery **(A)** on the 8th day after surgery, Dopples examination showed that the pulmonary veins were led into the left atrium, the internal diameter of the anastomosis was approximately 5 mm, the forward blood flow velocity of the left atrium of the pulmonary vein was approximately 2.1 m/s, and the pressure difference was 18 mmHg. The estimated pulmonary artery pressure was approximately 53 mmHg based on tricuspid regurgitant flow, indicating fast forward blood flow velocity of the anastomosis and high pulmonary artery pressure. Pericardial effusion (small amount), small regurgitation of the second and tricuspid valves, horizontal shunt of the great artery disappeared, no residual shunt, and residual obstruction were observed. **(B)** On the 23th day after surgery, ultrasound showed that pulmonary veins were led into the left atrium, the internal diameter of the anastomotic orifice was approximately 3 mm, the forward flow velocity of the anastomotic orifice was fast, the flow velocity was approximately 2.2 m/s, the pressure difference was 19mmHg, the horizontal shunt of the great artery disappeared, no residual shunt and obstruction were observed, atrial septal defect was observed, and the tricuspid valve regurgitant was observed. Pulmonary artery pressure is estimated to be 118 mmHg based on tricuspid regurgitation, pulmonary hypertension (severe). **(C)** On the 52th day after surgery, ultrasonography showed that the pulmonary veins were led into the left atrium, the internal diameter of the anastomosis was approximately 3.3 mm, the forward blood flow velocity was increased at approximately 3.0 m/s, the pressure difference was 36 mmHg, the atrial septal echo was lost approximately 2 mm, the pulmonary vein opening was small, the pulmonary vein was obstructive, and the tricuspid valve had a large amount of regurgitant flow. Pulmonary artery pressure was estimated to be approximately 100 mmHg based on tricuspid regurgitation, with pulmonary hypertension (severe) and reduced left ventricular systolic function.

After admission to the ICU, the patient was administered a course of cefoperazone sulbactam sodium and meropenem to enhance anti-infective measures, creatine sodium phosphate and myocardial peptide to nourish the myocardium, and hormones to reduce exudation. Three hours after surgery, chest and abdominal plain radiographs (Figure 2A) were reviewed, and the shape and size of the heart were normal. On the first day after surgery, the patient exhibited generalized edema. The left ventricular end-diastolic diameter was approximately 11 mm, with an EF of 63%. The patient was successfully weaned from ECMO support on the 4th day after surgery, but later on the 5th day after surgery, the chest drainage volume began to be excessive, and then intermittent turbidity appeared. A chylothoracotomy was contemplated, and the patient was placed on a water-only diet with intravenous nutritional support. On the 6th day after surgery, chest x-rays (Figure 2B) were re-examined, and the chest was successfully closed on the 7th day. Cardiac ultrasound (Figure 4A) was re-examined on the 8th day after surgery, indicating that the anterior blood flow rate of the anastomosis was 2.1 m/s. Chest x-rays (Figure 2C) indicated a right pneumothorax, which was considered to be caused by leakage of the chest catheter. Gastrointestinal nutritional support therapy was gradually given to milk protein hydrolyzed formula milk powder on the 11th day after surgery, and ventilator assistance was successfully withdrawn on the 21st day after surgery. On the 23rd day after surgery, chest x-rays (Figure 2D) indicated atelectasis of the right upper lung, and thoracocentesis was performed. During this period, the child gradually recovered, his daily intake was approximately 500 ml, urine volume was approximately 400 ml, and body swelling subsided. The patient was subsequently discharged from the ICU on the 30th day after surgery. On the 35th day after surgery, cardiac ultrasound (Figure 4B) and chest x-rays (Figure 3E) indicated a small amount of fluid in the right thoracic cavity. Thoracentesis was performed, and lung function was exercised by intermittent oxygen inhalation. On the 39th day after surgery, the child had shortness of breath, 55 breaths/min, positive trisocele sign, and BP of 73/36 mmHg. The child was promptly transferred to the ICU and initiated on non-invasive respiratory support with positive end-expiratory pressure (PEEP) of 4 cmH_2_O, oxygen concentration 60%, and flow rate 10 L/min, yet he exhibited labored breathing despite the non-invasive respiratory assistance. Additionally, the patient displayed cyanosis, and a venous blood gas check revealed the following: pH 7.082, PO_2_ 19.1 mmHg, PCO_2_ 109.7 mmHg, electrolyte potassium 7.33 mmol/L, HCT 30%, and Lac 12.8 mmol/L. Liver and kidney function and blood routine test were generally normal. Intravenous nutritional support and rehydration were provided with amino acids and glucose, with formula milk via nasal feeding. Additionally, spironolactone, a diuretic, was administered. Recombinant human brain natriuretic peptide was administered at 0.0075 µg/kg, resulting in improved cardiac function. After treatment, arterial blood gas analysis showed the following: pH 7.346, PO_2_ 57.6 mmHg, PCO_2_ 63.7 mmHg, electrolyte potassium 4.66 mmol/L, HCT 29%, and Lac 0.8 mmol/L. Chest x-rays (Figure 3F) were re-examined, intermittent prone ventilation was given, and sildenafil citrate tablets were given to reduce pulmonary artery pressure. On the 7th day of non-invasive respiratory assistance, due to worsening respiratory distress, with an RR of 65–68 breaths/min and cyanosis, the decision was made to proceed with tracheal intubation and ventilation again. The percutaneous oxygen saturation was 90%, and chest x-rays were re-examined (Figure 3G). Severe pulmonary hypertension persisted, prompting thoracentesis and insertion of a closed thoracic drainage tube.

In the following days, the patient received 152 ml of turbiditic fluid from a closed drainage tube in the left chest cavity daily. Cefoperazone and sulbactam were given to enhance anti-infection, as well as ambroxol to dilute sputum, and dexmedetomidine was continuously infused for sedation. Respiratory rate was maintained at approximately 36 breaths/min, with percutaneous oxygen saturation at 95%, HR at 123 beats/min, and BP at 74/39 mmHg. On the 6th day of the second ventilator-assisted breathing, the child had a gray face, three depression signs, and respiratory distress (RR 56 breaths/min, HR 154 beats/min, BP 65/36 mmHg). Venous blood gas analysis showed pH 7.207, PO_2_ 54.5 mmHg, and PCO_2_ 20.9 mmHg, when the oxygen concentration was 80%. The electrolyte potassium was 8.49 mmol/L, HCT was 33%, and Lac was 6.4 mmol/L. The patient exhibited critical values for liver, kidney function, and coagulation indexes, including creatinine 59 umol/L, uric acid 655umol/L, alanine aminotransferase 3,654 U/L, aspartate aminotransferase 10,200 U/L, total bilirubin 15.3 umol/L, lactate dehydrogenase >10,000 U/L, creatine kinase 1,215 U/L, prothrombin time (PT) 36 s, international normalized ratio (INR) 3.28, and activated partial thromboplastin time (APTT) 44.1 s. Preoperative chest x-rays (Figure 3H) and color Doppler echocardiography (Figure 4C) were performed to ascertain the child's current multiple organ failure (MOF) (heart, lung, liver, kidney), metabolic acidosis, hyperlactatemia, and hyperkalemia. This was done to determine the emergency treatment of pulmonary venous obstruction under cardiopulmonary bypass.

During the operation, fibrous tissue hyperplasia was observed at the anastomosis between the common pulmonary vein cavity and the left atrium. The hyperplastic tissue extended from the anastomosis into the common pulmonary vein cavity, reaching the openings of the left and right pulmonary veins, and the hyperplastic tissue was then removed. Part of the atrial septum was sutured continuously with 5–0 Prolene suture, and the size of the atrial septal defect of approximately 8 mm was preserved. Following the cessation of open circulation, necessitating the utilization of a temporary pacemaker, the pacing heart rate was 150 beats/min. Attempts to terminate the heart–lung machine were unsuccessful, and the child's condition remained critical, rendering it unfeasible to maintain an independent heart rate and blood pressure. ECMO assistance was considered, but the family decided to give up treatment after being given detailed information about the child's current condition.

## Discussion

At present, as more and more CPVA cases have been discovered, it is necessary to continue the research on this disease. For CPVA cases, early diagnosis and timely surgery are key to treatment. In the absence of prenatal diagnosis, it becomes more challenging to obtain the optimal time for surgery (9). Unfortunately, even if timely diagnosis and surgical repair are attempted, survival is not optimal and usually depends on the size of the venous junction (3). This is often lacking in patients with CPVA. Delay in surgery and treatment will inevitably lead to deterioration and death, or ECMO may be required, with treatment-related complications (5).

In newborns diagnosed with CPVA, the condition deteriorates rapidly due to deep hypoxia and severe acidosis, and if left untreated, most children die within a few days after birth. Therefore, early diagnosis of CPVA is essential for the success of surgery and treatment. In 2016, three patients diagnosed with CPVA were reported in Tennessee (10), and none of the three patients were found during the initial examination. Some patients had severe symptoms and were only found through imaging examination. Perez et al (3) pointed out that CPVA is similar to complete pulmonary venous ectopic drainage but requires early identification and differentiation of phenotypes, which is critical for the surgery and treatment of this rare disease. At present, the diagnosis of CPVA usually requires imaging. However, prenatal ultrasound is very difficult to diagnose CPVA, and it is very easy to miss or misdiagnose it as complete ectopic pulmonary venous drainage, thus affecting the optimal treatment time for patients. According to the International Pediatric and Congenital Cardiac Code, this is a different diagnosis from complete ectopic pulmonary venous drainage (11). When the ultrasound does not clearly show vertical venous drainage in the common lumen of the pulmonary vein or cannot trace the blood flow in the common lumen of the pulmonary vein or detect the end-diastolic pulmonary artery and the countercurrent of the aortic isthmus from early systolic to end-diastolic (12), these features help diagnose the disease. The diagnosis of CPVA should also be considered in clinical neonates with the following symptoms: deep cyanosis that does not change in response to oxygen therapy, refractory acidosis that cannot be cured after treatment, severe pulmonary venous congestion, and rapidly worsening disease changes.

Through the PubMed literature retrieval, a case review found that pulmonary lymphangiectasia (PL), spontaneous pneumothorax, chylothorax, and pneumomediastinum in many CPVA cases exist. PL is the most common non-cardiac-related presentation. This may be due to pulmonary congestion and an excess of pulmonary venous blood being shunted into the lymphatic channels without an obvious alternative decompression pathway. In addition, a significant expansion of the pulmonary lymphatic vessels helps to remove excess interstitial fluid from the lungs. After the birth of the fetus, placental circulation is interrupted; the newborn lung begins to breathe; the arterial catheter, intravenous catheter, and umbilical cord blood tube are disused; and complete pulmonary circulation appears. Pulmonary edema impairs gas exchange, and pulmonary vein obstruction inhibits the normal increase in pulmonary blood flow, leading to severe cardiopulmonary symptoms within minutes of birth. PL was secondary to increased right ventricular pressure and myocardial hypertrophy due to the gradual increase in pulmonary pressure. There is evidence that PL is associated with severe pulmonary venous obstruction, and PL may be an important survival risk factor in CPVA and other forms of pulmonary venous obstruction (2, 5, 13, 14). As reported in this case, chylothorax appeared in the early postoperative period (Day 4), during which the degree of chylothorax was slightly improved after treatment, but then the degree of chylothorax suddenly worsened. Despite the implementation of symptomatic measures and treatment, the condition remained unimproved and subsequently deteriorated. Upon review of the case, it was indicated that the blood flow velocity of the anastomotic orifice was accelerated during the cardiac ultrasound examination of the child, which coincided with the sudden worsening of the chylothorax condition. Therefore, we inferred that the degree of chylothorax was aggravated and that the possibility of anastomotic obstruction and occlusion should be monitored with vigilance at this time. Furthermore, the examination data should be considered in conjunction with this information to facilitate the formulation of a timely treatment plan.

Newborn hearts are more resistant to hypoxia than adult hearts, but severe hypoxia in critically ill newborns can inhibit systolic function and high-energy phosphate levels, resulting in slow recovery of systolic and diastolic function after ischemia arrest and reperfusion, which affects the recovery of cardiac function (15). In a case report of a child successfully treated with CPVA, it was pointed out that adverse preoperative symptoms (acidosis and cyanosis) may seriously impair the protective function of the newborn heart. However, the infant with these adverse conditions was urgently operated on within 7 h after birth, and the procedure was completed with a favorable prognosis (7). The patient we reported already had severe cyanosis, metabolic acidosis, hyperlactacidemia, respiratory failure, and heart failure upon admission. Although we had urgently conducted relevant examinations and arranged surgery (22 h after birth), the optimal operation time for safe operation tolerance and good prognosis had been missed for this patient. This has a great impact on his prognosis and recovery of cardiac function. Therefore, for children with these severe symptoms, early diagnosis and surgery are a key part of the successful treatment of children with CPVA. Despite the patient's successful initial surgical procedure, the postoperative examination data indicated that the patient's condition remained critical, necessitating close observation and prompt adjustments to the treatment plan as needed. The echocardiography we reviewed on the 8th day after surgery indicated that the forward blood flow velocity of the anastomosis was 2.1 m/s, and the echocardiography data we reviewed later showed that the forward blood flow velocity of the anastomosis was 2.2 m/s. We did not promptly alert to the possibility of pulmonary venous obstruction, which affected the best time for the correction of pulmonary venous obstruction. At that time, the disease of the child had developed to the stage of MOF, the ability to tolerate surgery was very poor, and the best opportunity for correction was missed, resulting in irreversible disease. We consider the factors that contributed to the development of MOF in our patient: the fragile state of the organism due to severe preoperative metabolic acidosis (manifested by severe hyperlactatemia); the load on the heart and body from the prolonged duration of the extracorporeal circulation; the shock of the secondary surgery; and the postoperative chylothorax. In our opinion, although the surgical repair was technically successful, the above factors and the missed timing of targeted therapy ultimately led to irreversible MOF. For CPVA patients who underwent direct cardiac repair after venous malformation drainage, the surgeon should always be alert to the possibility of pulmonary vein obstruction or even occlusion, timely review of ultrasound, and pay attention to the anastomotic blood flow velocity. When the anastomotic blood flow velocity accelerates, the degree of chylothorax suddenly worsens, and the patient's respiratory function deteriorates, the possibility of pulmonary vein obstruction should be timely considered. It is imperative to devise a surgical plan for treatment at the earliest opportunity to ensure that the optimal window for correction is not missed, which would otherwise jeopardize the success of the treatment.

## Conclusion

Despite ongoing medical advances, the prognosis for CPVA remains poor, and early diagnosis and surgical treatment remain challenging. In the fetal period, a clear diagnosis of CPVA in the prenatal period is of great significance to the prognosis. The establishment of a comprehensive management system comprising multidisciplinary experts in ultrasound imaging, obstetrics, cardiac surgery, and other relevant disciplines, and the transfer of the patient to the cardiac surgery department for immediate surgical intervention following pregnancy, represents a crucial step in achieving a favorable outcome. The critical time window for postnatal intervention is within 24 h of birth. Subsequent to the surgical procedure, it is imperative to closely monitor the patient's imaging and other examination data for any alterations. We suggest that the cardiac ultrasound testing intervals be assessed weekly in the early postoperative period, fortnightly after 1 month, and monthly after 3 months. It is still necessary to always be alert to the occurrence of pulmonary vein obstruction and implement targeted therapies in time according to the changes in patients’ conditions in order to achieve good results for patients. The patient we reported this time underwent two operations successively. Although the outcome was not optimistic, the changes in examination data and the treatment plan made for the patient during the evolution of the patient's condition can be used as a reference for you, hoping to provide some help in improving the treatment of CPVA patients.

## Data Availability

The original contributions presented in the study are included in the article. Further inquiries can be directed to the corresponding author/s.

## References

[1] Nakamura Y Miyaji K Miyata Y Kitagawa A. An extremely rare variant of pulmonary venous atresia. Ann Thorac Surg. (2016) 101(6):2382–4. 10.1016/j.athoracsur.2015.08.085 27211953

[2] Fragata J Magalhães M Baquero L Trigo C Pinto F Fragata I Partial anomalous pulmonary venous connections: surgical management. World J Pediatr Congenit Heart Surg. (2013) 4(1):44–9. 10.1177/2150135112460250 23799753

[3] Perez M Kumar TKS Briceno-Medina M Alsheikh-Ali M Sathanandam S Knott-Craig CJ. Common pulmonary vein atresia: report of three cases and review of the literature. Cardiol Young. (2016) 26(4):629–35. 10.1017/S1047951115002334 26510606

[4] Vaideeswar P Tullu MS Sathe PA Nanavati R. Atresia of the common pulmonary vein–a rare congenital anomaly. Congenit Heart Dis. (2008) 3(6):431–4. 10.1111/j.1747-0803.2008.00225.x 19037984

[5] Thourani VH Kirshbom PM Kanter KR Simsic J Kogon BE Wagoner S Venoarterial extracorporeal membrane oxygenation (VA-ECMO) in pediatric cardiac support. Ann Thorac Surg. (2006) 82(1):138–45. 10.1016/j.athoracsur.2006.02.011 16798204

[6] Mas C Cochrane A Menahem S Knight B. Common pulmonary vein atresia: a diagnostic and therapeutic challenge. Pediatr Cardiol. (2000) 21(5):490–2. 10.1007/s002460010119 10982717

[7] Suzuki T Sato M Murai T Fukuda T. Successful surgical repair of common pulmonary vein atresia in a newborn. Pediatr Cardiol. (2001) 22(3):255–7. 10.1007/s002460010217 11343159

[8] YanPeng S Bing Z. A case of prenatal ultrasound misdiagnosis of complete ectopic drainage of the pulmonary veins due to pulmonary venous colluminal atresia. Chin J Perinat Med. (2018) 21(3):181–3. 10.3760/cma.j.issn.1007-9408.2018.03.007

[9] Glenn T Honold J Printz BF Mueller D. Common pulmonary vein atresia. Cardiol Young. (2022) 32(4):668–70. 10.1017/S1047951121003565 34486517

[10] Anderson RH. Atresia of the common pulmonary vein: the importance of phenotypic recognition. Cardiol Young. (2016) 26(4):636–7. 10.1017/S1047951115002450 26555433

[11] Walsh MJ Ungerleider RM Aiello VD Spicer D Giroud JM. Anomalous pulmonary venous connections and related anomalies: nomenclature, embryology, anatomy, and morphology. World J Pediatr Congenit Heart Surg. (2013) 4(1):30–43. 10.1177/2150135112458439 23799752

[12] Nagasawa H Hirata K. Specific echocardiographic findings useful for the diagnosis of common pulmonary vein atresia. Pediatr Rep. (2015) 7(4):6228. 10.4081/pr.2015.6228 26734125 PMC4689990

[13] Yamada S Hisaoka M Wang KY Ding Y Guo X Shimajiri S Total anomalous pulmonary vein drainage: report of an autopsy case associated with atresia of the common pulmonary vein and left superior pulmonary vein. Pathol Int. (2011) 61(2):93–8. 10.1111/j.1440-1827.2010.02617.x 21255186 PMC3047006

[14] AlTeneiji M Brundler MA Noseworthy M Kurek KC. Unilateral congenital pulmonary lymphangiectasis presenting with pneumothorax and an NRAS variant. Pediatr Pulmonol. (2021) 56(7):2374–6. 10.1002/ppul.25401 33852777

[15] Hammon JW Jr. Myocardial protection in the immature heart. Ann Thorac Surg. (1995) 60(3):839–42. 10.1016/0003-4975(95)00573-4 7677543

